# Multi-omics analysis of the anti-cancer effects of curcumol in endometrial carcinoma

**DOI:** 10.3389/fphar.2025.1565959

**Published:** 2025-07-03

**Authors:** Jumin Xie, Ying Zhu, Haozhen Bai, Zean Song, Shukai Qiu, Youyi Xie, Shuqiong Wan

**Affiliations:** ^1^ Hubei Key Laboratory of Renal Disease Occurrence and Intervention, Medical school, Hubei Polytechnic University, Huangshi, Hubei, China; ^2^ Department of Gynecology, Huangshi Central Hospital, Affiliated Hospital of Hubei Polytechnic University, Huangshi, Hubei, China

**Keywords:** anticancer, curcumol, endometrial carcinoma, machine learning, multi-omics analysis

## Abstract

**Background:**

Endometrial carcinoma (EC) is one of the most common gynecologic malignancies, with increasing global morbidity and mortality rates. Curcumol, a sesquiterpenoid hemicrystalline compound, exhibits notable pharmacological effects, including anticancer, anti-inflammatory, and antiviral properties. This study aims to explore the molecular mechanisms through which curcumol exerts its effects in the treatment of EC.

**Methods:**

Network pharmacology, data mining and machine learning were used to integrate curcumol and EC targets. R and online databases were applied to screen core targets. The core targets were verified by molecular docking, molecular dynamics simulation, ceRNA network regulation, clinical sample staining, and immunoinfiltration analysis.

**Results:**

Progesterone Receptor (PGR) and Ribosomal protein S6 kinase (RPS6KA1) were identified as two core targets in the cancer risk prognostic model. Survival analysis indicated that high expression of PGR and RPS6KA1 is associated with prolonged survival in patients with EC. The HPA validation confirmed the low expression of PGR and high expression of RPS6KA1 in EC tissues. Molecular docking and simulation confirmed strong binding affinities between curcumol and the PGR and RPS6KA1 targets. Myc-associated zinc finger protein (MAZ) was a regulator of both PGR and RPS6KA1. Additionally, KCNQ1OT1 and chr22-38_28785274–29006793.1 were found to jointly regulate PGR and RPS6KA1 through various miRNAs, contributing to the pathogenesis of EC.

**Conclusion:**

Through multi-omics analysis, we conclude that curcumol exerts its anticancer effects primarily through the core targets PGR and RPS6KA1 in the treatment of EC.

## 1 Introduction

Endometrial carcinoma (EC) is one of the most common gynecological malignancies and the sixth most common cancer among women (Sung et al., 2021). The age-standardized mortality rate (ASMR) was 2.1 per 100,000 women. In 2022, there were 420,242 new cases, representing 2.1% of all cancers, and 97,704 deaths, or about 1% (Bray et al., 2024). The lifetime risk of EC for women is approximately 3%, with a median age at diagnosis of 61 years (Crosbie et al., 2022).

EC can be divided into three histological subtypes: endometrioid carcinoma, serous carcinoma, and clear cell carcinoma (Urick and Bell, 2019; Vermij et al., 2020). Based on pathogenesis and biological behavior, EC is classified into estrogen-dependent (Type I) and non-estrogen-dependent (Type II) types. Type I accounts for approximately 80%–90% of cases, predominantly comprising endometrioid carcinoma, with the remaining cases being mucinous adenocarcinoma. Type II accounts for about 10%–20% of cases and includes non-endometrioid subtypes such as serous carcinoma, clear cell carcinoma, undifferentiated carcinoma, and carcinosarcoma (Makker et al., 2021; Murali et al., 2014).

The most common early clinical manifestation of EC is postmenopausal bleeding, although only 5%–10% of women with postmenopausal bleeding will develop cancer (Crosbie et al., 2022). Risk factors for EC are primarily linked to prolonged, unopposed estrogen exposure, including estrogen replacement therapy, early menarche, late menopause, tamoxifen use, nulliparity, infertility or ovulatory disorders, polycystic ovary syndrome, and elevated postmenopausal estrogen levels (Braun et al., 2016; Colombo et al., 2016). Additional risk factors include increasing age, obesity, hypertension, diabetes, and hereditary non-polyposis colorectal cancer (Mahdy et al., 2025).

The primary treatment for EC is hysterectomy with bilateral salpingo-oophorectomy. Radiotherapy and chemotherapy also play roles in adjuvant therapy, including pelvic external beam radiation, vaginal brachytherapy, and combination chemoradiotherapy (van den Heerik et al., 2021). Low-risk to moderate endometrial hyperplasia can be managed with non-surgical options. Survival is generally determined by disease stage and histology, with most patients in stage I and II having a favorable prognosis (Braun et al., 2016; Colombo et al., 2016; Mahdy et al., 2025). Although early-stage EC is often diagnosed with a good prognosis, it remains one of the few cancers with an increasing mortality rate, with recurrence rates ranging from 2% to 15% in early-stage patients and possibly as high as 50% in high-risk individuals (Brooks et al., 2019; Riedinger et al., 2022).

Therefore, there is an urgent need to develop more effective therapeutic agents for EC to improve patient survival, quality of life, and reduce treatment costs and the economic burden on patients (Feng et al., 2024; Li et al., 2025). One objective of this study is to identify small chemical molecules with potential therapeutic effects on EC from natural products and explore their therapeutic value.

Curcumol, a natural guaiacoid sesquiterpenoid semi-crystalline compound with biological activity, can be isolated from *Curcuma longa L*., *Curcumae rhizoma*, and other plants. Studies have shown that natural guaiacoid sesquiterpenoids exhibit potent antibacterial, anti-inflammatory, anticancer, and neuroprotective effects (Guo et al., 2023; Wang et al., 2020). In recent years, curcumol has demonstrated pharmacological properties such as anti-inflammatory, antiviral, anticonvulsant effects, as well as the ability to protect the liver and positively impact the treatment of various solid tumors, liver diseases, inflammatory disorders, and infectious diseases (Hashem et al., 2021; Wei et al., 2019; Wu and Wang, 2024; Zhai et al., 2024).

In clinical practice, endometriosis is recognized as a precursor lesion for several malignancies, including endometriosis-related cancers (Kajiyama et al., 2019; Terzic et al., 2021). Curcumol effectively reduces inflammatory cytokine levels released by ectopic endometrial stromal cells by inhibiting the JAK2/STAT3 signaling pathway, thereby suppressing cell proliferation, migration, and reducing the volume of ectopic lesions (Wang et al., 2022). However, the precise target and mechanism of curcumol's anti-EC effect remain unclear.

Therefore, this study aims to explore the potential pharmacological effects of curcumol in the treatment of EC through network pharmacology, multi-omics technologies, and molecular docking validation. The aim is to elucidate the molecular mechanisms underlying curcumol’s effects on EC and provide valuable insights for the development of small-molecule compounds as therapeutic agents for EC.

## 2 Materials and methods

### 2.1 Target collection of curcumol

In the BATMAN-TCM2.0 database (http://bionet.ncpsb.org.cn/batman-tcm/#/home) (Kong et al., 2024) and the HIT2.0 database (http://www.badd-cao.net:2345/) (Yan et al., 2022), the relevant targets for curcumol were identified using keyword searches. The Isomeric SMILES of curcumol were retrieved from the PubChem database (https://pubchem.ncbi.nlm.nih.gov/) (Kim et al., 2023) and used in the SwissTargetPrediction database (http://swisstargetprediction.ch/) (Daina et al., 2019), TargetNet database (http://targetnet.scbdd.com/) (Yao et al., 2016), and the SuperPred database (https://prediction.charite.de/) (Gallo et al., 2022) to collect potential targets for curcumol.

In the SwissTargetPrediction database, *Homo sapiens* was selected as the species, and targets with a “Probability >0.01″were screened. In the TargetNet database, targets with an “AUC ≥0.7″and “Probability >0.01” were selected. For the SuperPred database, targets with a “Probability >60%” were filtered. The UniProt database (https://www.uniprot.org/) (The UniProt Consortium, 2023) was used to convert UniProt IDs to gene names. The target sets from these five databases were compiled to obtain a comprehensive list of curcumol targets, which were then visualized using the bioinformatics platform (https://www.bioinformatics.com.cn/) (Tang et al., 2023). Targets collection date was up to 2 December 2024.

The curcumol targets extracted from the five databases were summarized and integrated. Specifically, deduplication was performed on the target data to avoid double-counting. For data with discrepancies or conflicts, a comprehensive assessment was conducted, considering factors such as the credibility of the database predictions, update frequency, and consistency with other databases (Xie et al., 2022).

### 2.2 EC target collection

TCGA RNA-Seq HTSeq-FPKM data for Uterine Corpus Endometrial Carcinoma (UCEC) were downloaded using the Sangerbox platform (http://sangerbox.com/home.html) (Shen et al., 2022). The dataset included 579 samples, consisting of 35 control and 544 tumor samples. ID conversion was performed, and the dataset was standardized. Differential expression analysis was conducted using the “limma” package (version 3.40.6) in R software. Differentially expressed genes (DEGs) were identified using a p-value <0.05 and |log_2_ fold change| > 1 as the cutoff criteria. Heatmaps and volcano plots were generated to visualize the results. Data collection date was up to 3 December 2024.

### 2.3 GO and KEGG enrichment analysis of EC-curcumol targets

Curcumol targets and UCEC-associated targets were intersected to identify common targets via the bioinformatics platform. The DAVID database (https://david.ncifcrf.gov/) (Sherman et al., 2022) was used for Gene Ontology (GO) functional analysis and KEGG pathway enrichment analysis, with the species limited to “*H. sapiens*.” For GO analysis, the top 20 terms based on p-value <0.05 for Biological Process (BP), Cellular Component (CC), and Molecular Function (MF) were selected. A Sankey diagram was generated using the CNSknowall platform (https://www.cnsknowall.com/#/HomePage). Additionally, the KEGG pathway terms with p-value <0.05 were imported into the CNSknowall platform to generate a KEGG pathway string diagram.

### 2.4 Construction and evaluation of cancer risk prognostic model

To further assess the impact of EC-curcumol targets on cancer prognosis, the “survival” package in R was used to integrate survival time, survival status, and gene expression data. The prognostic significance of the intersection targets was evaluated using the Cox proportional hazards method. Univariate Cox proportional hazards regression analysis was performed, and forest plots were generated (Tay et al., 2023). Genes with p-value <0.05 in the univariate analysis were considered prognostic genes for subsequent analysis.

To prevent overfitting, the “glmnet” package in R was used to integrate survival time, survival status, and gene expression data, followed by regression analysis using the LASSO -Cox method (Wang et al., 2019). Multivariate survival analysis was conducted using the “survival” package in R. The data, including survival time, survival status, and five features, were integrated. The prognostic significance of these features in 539 samples was assessed using the Cox method. Patients were stratified into two groups based on the 50% percentile of the risk score. The “survfit” function from the “survival” package was then used to analyze the prognostic differences between the two groups. The significance of prognostic differences between groups was evaluated using the log-rank test, and Kaplan-Meier survival analysis was performed (May, 2009).

Subsequently, combining survival time and survival status data, ROC analysis was carried out using the “pROC” package in R (version 1.17.0.1) (Lorent et al., 2014). ROC curves for 1, 3, and 5 years were plotted to calculate the area under the curve (AUC) and evaluate the accuracy of the prognostic model (Nahm, 2022). Additionally, the risk score changes from low to high were visualized, and the relationship between follow-up time, survival status, gene expression, and risk score changes was analyzed (Xie et al., 2025).

### 2.5 Core gene survival analysis

The core genes used to construct the prognostic models were uploaded to the Sangerbox website for survival curve analysis. This analysis examines the impact of these core genes on patient outcomes over time.

### 2.6 Core protein expression profile

Core protein expression profile between normal and EC tissues were analyzed using the Human Protein Atlas (HPA) database (https://www.proteinatlas.org/) (Uhlén et al., 2015). The stained area was quantified using ImageJ Pro Plus 6 software and statistically analyzed with GraphPad Prism 8.0 software.

### 2.7 Gene set enrichment analysis (GSEA)

The “org.Hs.e.g.,.db” package in R (Version 4.3.3) was used to determine the biological significance and function of core genes through the “c2. cp.kegg.Hs.symbols” file. After 100 permutations, genes were considered significantly enriched if they met the thresholds of p-value <0.05 and FDR <0.25. The top five pathways most enriched in the high-expression and low-expression groups were selected and visualized using the “enrichplot” package. A positive Enrichment Score (ES) indicates activation of the gene set, while a negative ES suggests suppression of the gene set.

### 2.8 Molecular docking and molecular dynamics simulation

The core targets were retrieved from the UniProt database and filtered by “Reviewed” and “Human” entries. The corresponding target entries were then copied and searched in the RCSB PDB database (https://www.rcsb.org/) (Berman et al., 2000). Based on *H. sapiens*, full-length sequences, unique ligands, and high-resolution methods, the corresponding target protein crystal structures were selected.

The 2D structure of curcumol was obtained from the PubChem database. Initially, Chem3D software was used to convert the structure into mol2 format. For the target proteins, water molecules and extraneous residues were removed using PyMOL (version 2.2) (http://www.pymol.org/2/) (Xie et al., 2022), missing atoms were supplemented with PyMOL, and the structures were converted to pdb format. Both the target proteins and the curcumol molecule were processed with AutoDockTools 1.5.7 and converted to pdbqt format.

Molecular docking between the curcumol molecule and the core targets was performed using AutoDock Vina (https://vina.scripps.edu/) (version 1.5.7) (Morris et al., 2009; Trott and Olson, 2010). Complexes with low binding energy and favorable conformations were selected for visualization in PyMOL to assess hydrogen bonds.

Additionally, 2D molecular docking interactions were analyzed using the PROTEINS PLUS web tool (https://proteinsplus/) (Schöning-Stierand et al., 2020). Strong interactions between the core targets and curcumol were identified when the affinity score was ≤ −5.0 kcal/mol (Shamsol Azman et al., 2023).

Molecular dynamics (MD) simulations were conducted using GROMACS (Version 2020.6) (Arshia et al., 2021). The Amber GAFF2 force field was used to model the behavior of curcumol molecule, while the FF14SB protein force field was applied for the molecular dynamic simulation (He et al., 2020; Maier et al., 2015). The binding complex was immersed in a 10 × 10 × 10Å cubic box filled with TIP3P water molecules and neutralized with Na^+^ and Cl^−^ ions.

The energy of the binding complex was minimized using the steepest descent method followed by the conjugate gradient method. The thermodynamic temperature of the binding complex was then gradually increased from 0 K to 310 K under constant volume and uniform heating. A 200 ps NVT (constant Number of particles, Volume, and Temperature) simulation was performed after the binding complex stabilized at 310 K (Xie et al., 2025).

After pre-equilibration, an extended molecular dynamics simulation of 100 ns was conducted using the Nosé-Hoover algorithm-based Parrinello-Rahman constant-pressure barostat and V-rescale thermal bath method. Following the simulation, the binding complex underwent periodic corrections, and the Root Mean Square Fluctuation (RMSF), Root Mean Square Deviation (RMSD), and Radius of Gyration (Rg) of the curcumol-target complex were calculated (Xie et al., 2025). Visualization was generated using DuIvyTools (https://duivytools.readthedocs.io/en/latest/DIT.html).

### 2.9 Construction of regulatory network for core targets

Transcription factors associated with the core target were predicted using the ChEA3 database (https://maayanlab.cloud/chea3/) (Keenan et al., 2019), with the following filter conditions: ENCODE database selection and FET p-value <0.05. miRNAs related to the core targets were predicted using the TargetScan database (https://www.targetscan.org/vert_80/) (McGeary et al., 2019), with the selection criteria: PCT >0.5. Long non-coding RNAs (lncRNAs) were predicted using the DIANA-LncBase V2 database (http://carolina.imis.athena-innovation.gr/diana_tools/web/index.php) (Paraskevopoulou et al., 2016), with the threshold set to ≥0.999. Finally, the “TF-core target” network and the “lncRNA-miRNA-mRNA” network were constructed using Cytoscape 3.9.0 software (Shannon et al., 2003).

### 2.10 Immunoinfiltration analysis

Immune cell infiltration was estimated using the “CIBERSORT” algorithm (https://cibersortx.stanford.edu) with 1,000 permutations through R (Version 4.3.3). Samples with a “CIBERSORT” p-value <0.05 were filtered. The correlation between core genes and 22 types of infiltrating immune cells was then analyzed. Finally, the results were visualized using the R packages “reshape2” and “ggpubr”.

## 3 Results

### 3.1 Curcumol targets

**FIGURE 1:**
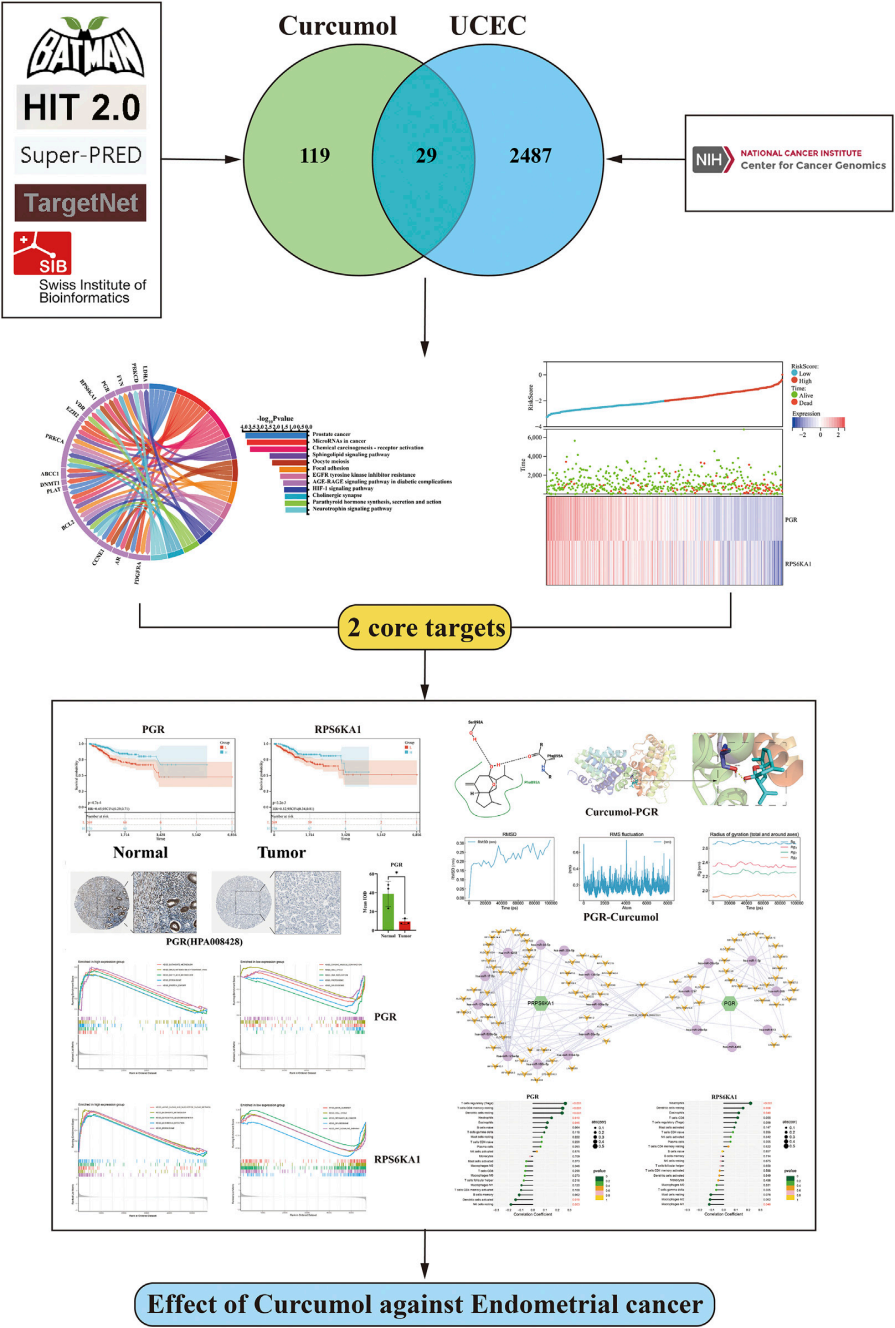
Flowchart of this study.

The flowchart of this study is shown in Figure 1.

**FIGURE 2:**
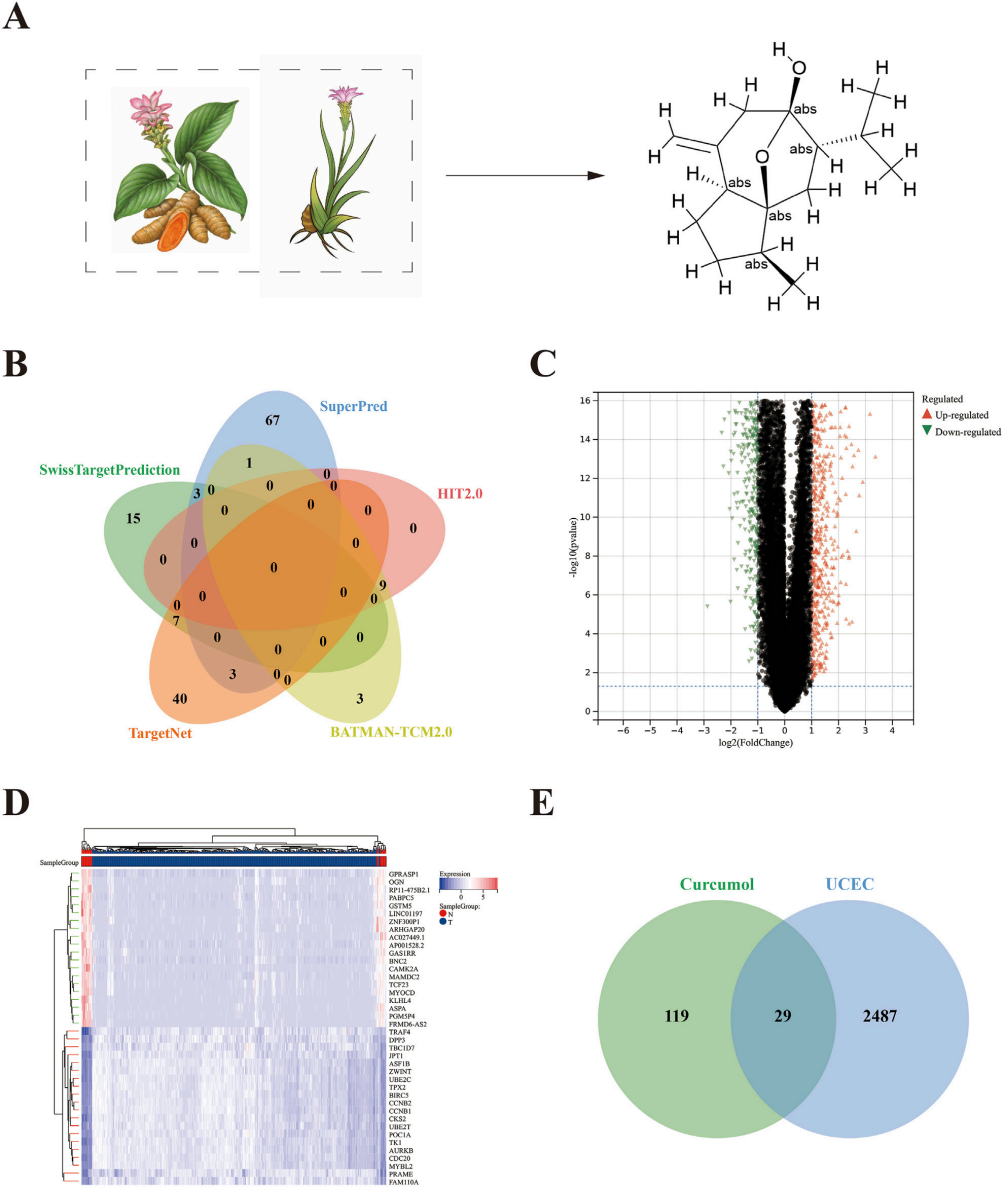
Identification of curcumol targets and DEGs in endometrial carcinoma. **(A)** Chinese medicinal herbs (*Carcuma longa*, *Curcuma zedoary*) containing curcumol and the molecular structure of curcumol. **(B)** The targets of curcumel were retrieved from five databases: BATMAN-TCM 2.0, HIT 2.0, SwissTargetPrediction, TargetNet, and SuperPred. **(C)** Volcano plot of DEGs. **(D)** Heatmap of DEGs. **(E)** Venn diagram of overlapping targets of curcumol and EC.

Curcumol is primarily extracted from Turmeric and Curcuma species, and its molecular structure is shown in Figure 2A. A total of 148 curcumol targets were identified from the following databases: 13 targets from the BATMAN-TCM2.0, 9 targets from the HIT2.0, 25 targets from the SwissTargetPrediction, 50 targets from the TargetNet, and 74 targets from the SuperPred. These targets were combined to yield 148 unique curcumol targets (Figure 2B).

### 3.2 DEGs of EC

Differential expression analysis was performed using the “limma” package in R software, resulting in 2,516 DEGs, including 1107 upregulated genes and 1409 downregulated genes (Figures 2C,D).

### 3.3 GO and KEGG enrichment analysis

**FIGURE 3:**
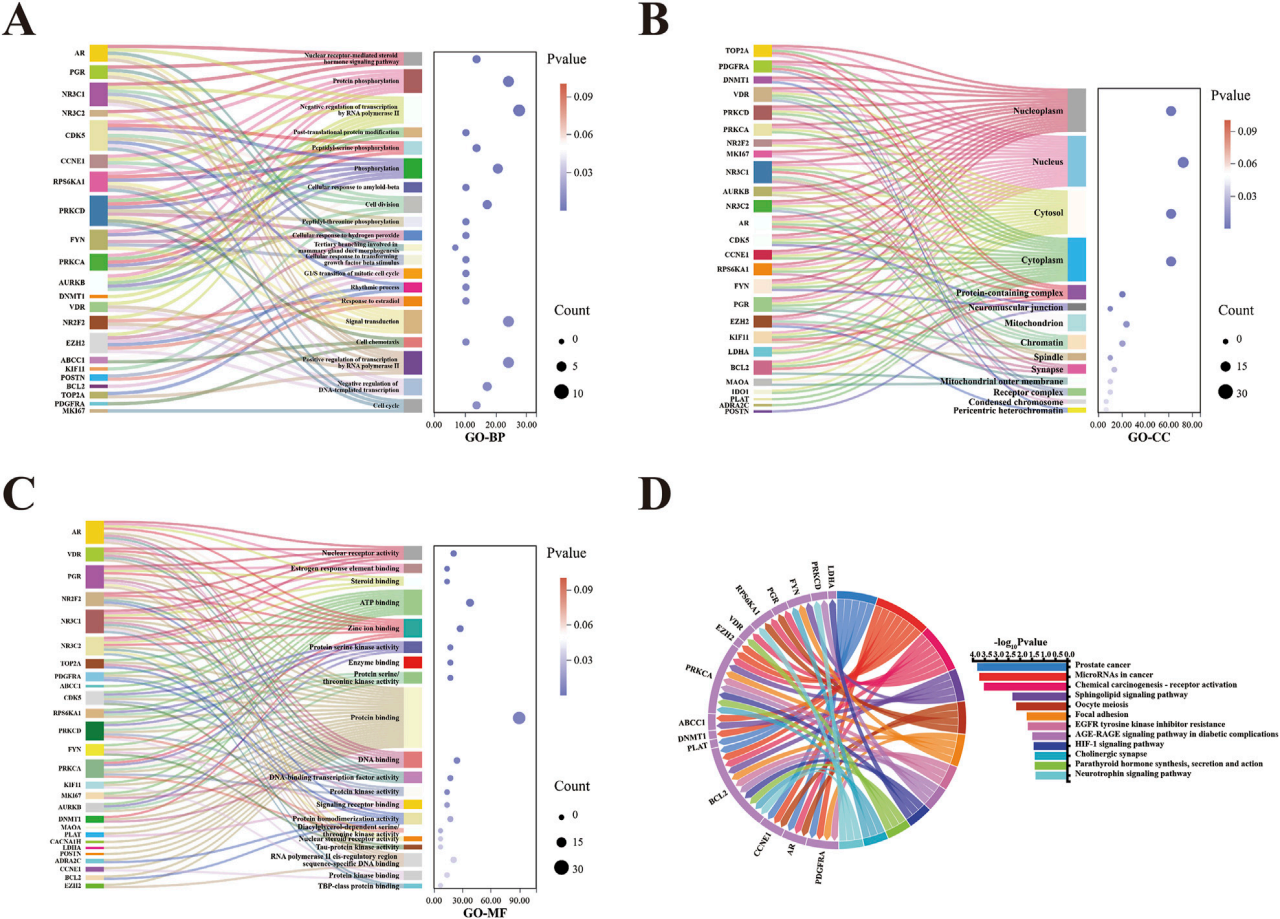
GO and KEGG enrichment analysis of 29 curcumol-EC targets. **(A–C)** BP, CC, and MF of curcumol-EC targets shown in a Sankey diagram. **(D)** Chord diagram of KEGG enrichment analysis for curcumol-EC targets.

The intersection of the 148 curcumol targets and the 2516 EC targets revealed 29 common targets (Figure 2E). GO enrichment analysis, including biological process (GO-BP), cellular component (GO-CC), and molecular function (GO-MF) categories, identified 70, 17, and 29 significant terms, respectively. The top 20 most significant pathways or terms (p-value <0.05) were used to construct Sankey plots (Figures 3A–C).

The results indicated that the BP associated with these targets were primarily related to the nuclear receptor-mediated steroid hormone signaling pathway, protein phosphorylation, and negative regulation of transcription by RNA polymerase II. In terms of CC, the targets were predominantly localized in the nucleoplasm, nucleus, and cytosol. MF analysis revealed that the target functions were significantly concentrated in nuclear receptor activity, estrogen response element binding, and steroid binding.

KEGG pathway enrichment analysis identified 20 significant signaling pathways, and the pathways with a p-value <0.05 were selected for visualization (Figure 3D). The key signaling pathways involved in curcumol’s anti-cancer effect on EC included microRNAs in cancer, sphingolipid signaling, oocyte meiosis, and the HIF-1 signaling pathway, among others.

### 3.4 Cancer risk prognostic model

**FIGURE 4:**
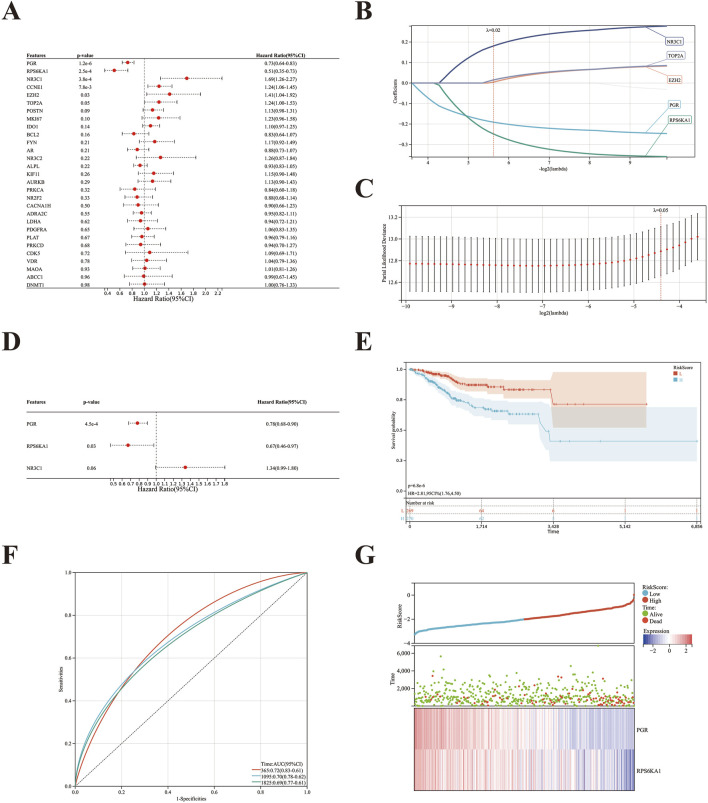
Construction and validation of risk prognostic model of EC. **(A)** Six prognostic genes were initially identified using univariate Cox regression analysis (P < 0.05). **(B)** The optimal parameter (λ) was selected based on the -log2(λ) sequence generation coefficient profile in the LASSO model. **(C)** LASSO regression analysis identified five prognostic genes. **(D)** Multivariate Cox analysis revealed three prognostic genes. **(E)** Kaplan-Meier survival curves for high- and low-risk groups. **(F)** ROC curves for overall survival at 1, 3, and 5 years of the prognostic model. **(G)** The relationship between the survival status of EC patients and the risk score from the prognostic model, along with changes in gene expression levels as the risk score increased.

A univariate regression analysis was performed on the 29 target genes of Curcumol-EC using the survival package in R software. Six genes were found to be significantly correlated with the overall survival (OS) of EC (P < 0.05), namely, PGR, RPS6KA1, NR3C1, CCNE1, EZH2, and TOP2A (Figure 4A). To prevent overfitting, LASSO-Cox regression analysis was applied to these six genes, with 10-fold cross-validation and a lambda value of 0.0204. As a result, five genes—PGR, RPS6KA1, NR3C1, EZH2, and TOP2A—were retained (Figures 4B,C).

Subsequently, a multivariate Cox regression analysis was performed on the five genes selected by LASSO-Cox regression. Three genes—PGR, RPS6KA1, and NR3C1—were identified, with PGR and RPS6KA1 confirmed as independent prognostic factors. A prognostic model for EC was constructed based on these two genes (Figure 4D). Patients were stratified into high- and low-risk groups using the median risk score as the cutoff. Survival analysis revealed that patients in the low-risk group had significantly better survival compared to those in the high-risk group, with a substantial prognostic difference (P = 6.8e-6) (Figure 4E).

The AUC of the ROC curve for OS prediction at the first, third, and fifth years was 0.72, 0.70, and 0.69, respectively (Figure 4F). Patients were ranked based on their risk scores, from low to high, and a risk score graph was generated. A plot of risk scores and survival time was also created, showing that as the risk score increased, the expression levels of PGR and RPS6KA1 decreased, patient survival time shortened, and the mortality rate significantly increased (Figure 4G).

### 3.5 Survival curve

**FIGURE 5:**
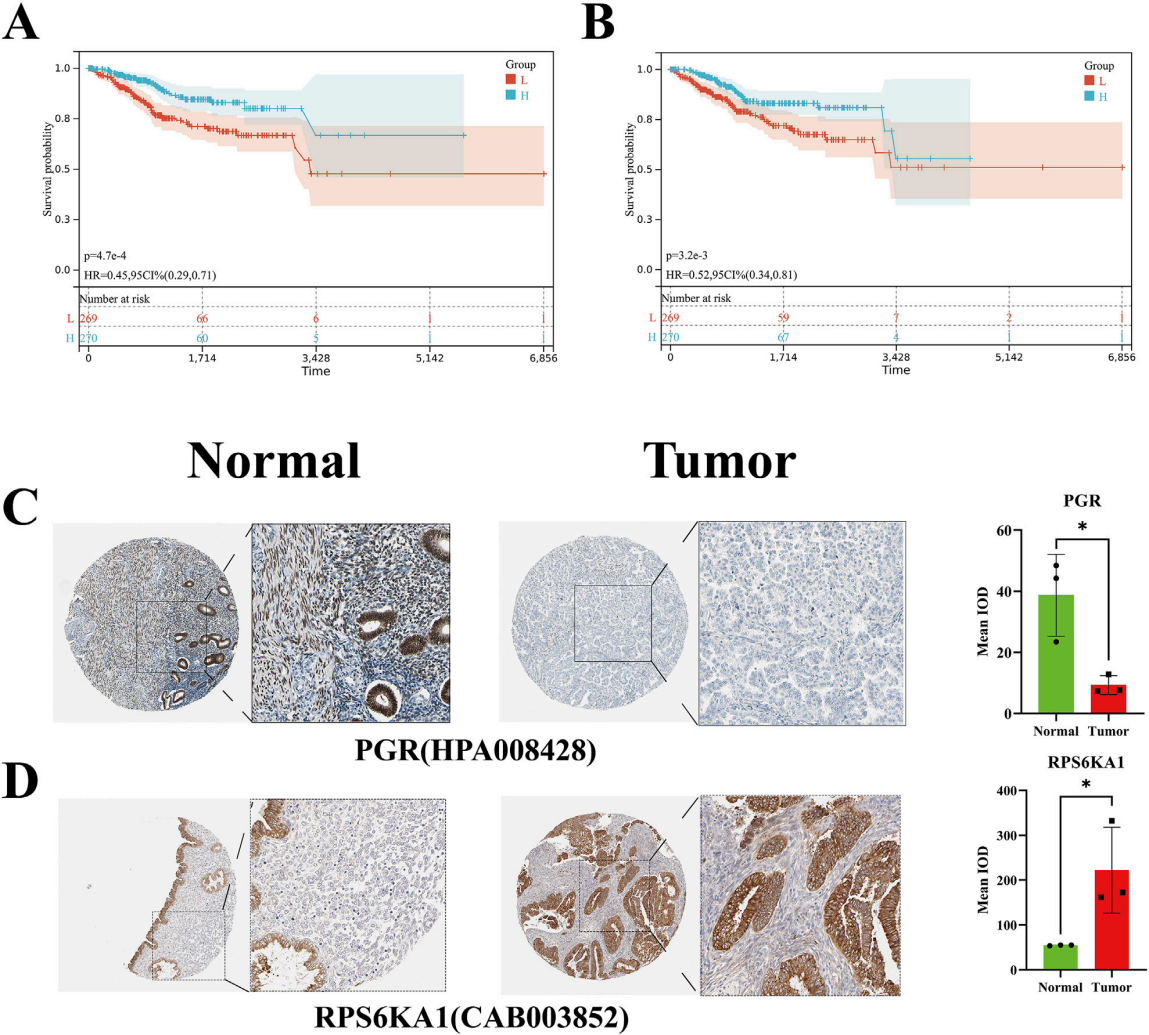
Survival curve and HPA validation. **(A,B)** Survival curve of PGR and RPS6KA1. **(C,D)** Immunohistochemical staining and statistical analysis of PGR and RPS6KA1 in normal endometrial and EC tissues.

On the SangerBox website, EC was defined as the disease of interest, and a survival curve was generated. The results indicated that the hazard ratios for PGR and RPS6KA1 were both less than 1, suggesting that promoting the expression of these genes may prolong survival in patients with EC (Figures 5A,B).

### 3.6 HPA validation

The immunohistochemical staining data for normal endometrial and EC tissues were obtained from the HPA database. The antibody used for PGR detection was HPA008428. The immunohistochemical results and statistical analysis revealed that, compared to normal tissues, EC tissues exhibited weaker staining, suggesting a reduced expression of PGR protein in EC tissues, with the difference being statistically significant. The antibody used for RPS6KA1 detection was CAB003852. The immunohistochemical results and statistical analysis showed that, in contrast to normal tissues, EC tissues displayed stronger staining, indicating an elevated expression of RPS6KA1 protein in EC tissues, with the difference being statistically significant (Figures 5C,D).

### 3.7 GSEA results

**FIGURE 6:**
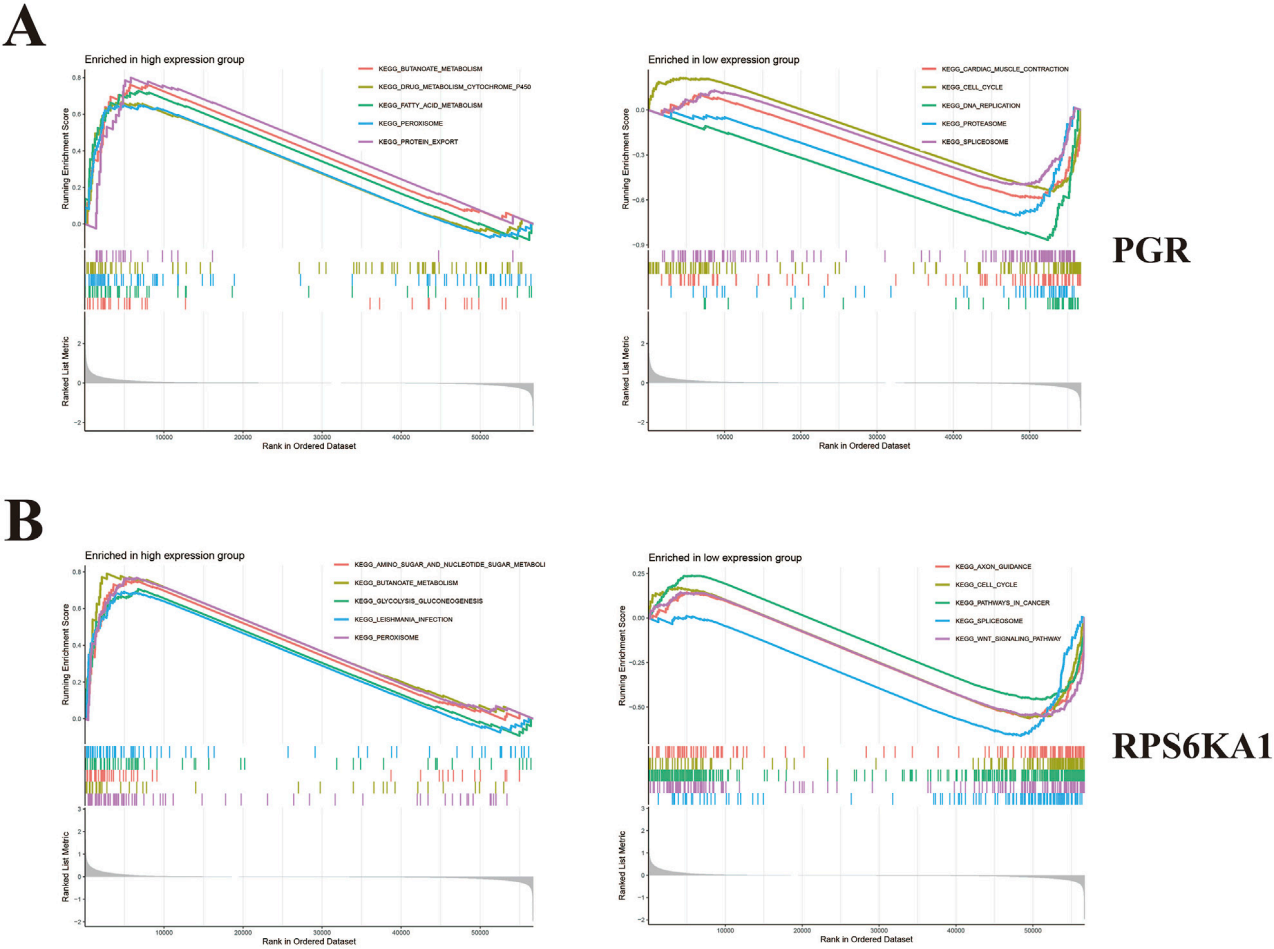
GSEA enrichment analysis. **(A)** Five pathways with significantly upregulated PGR and five pathways with significantly downregulated PGR. **(B)** Five significantly upregulated pathways and five significantly downregulated pathways in RPS6KA1.

GSEA enrichment analysis revealed that PGR was primarily upregulated in pathways related to butanoate metabolism, drug metabolism (cytochrome P450), fatty acid metabolism, peroxisome function, and protein export, whereas it was downregulated in pathways involving cardiac muscle contraction, the cell cycle, DNA replication, the proteasome, and the spliceosome (Figure 6A).

RPS6KA1 was primarily upregulated in amino sugar and nucleotide sugar metabolism, butanoate metabolism, glycolysis and gluconeogenesis, Leishmania infection, and peroxisome function, while it was downregulated in pathways related to axon guidance, the cell cycle, pathways in cancer, the spliceosome, and Wnt signaling (Figure 6B).

### 3.8 Molecular docking and molecular dynamics simulation validation

**FIGURE 7:**
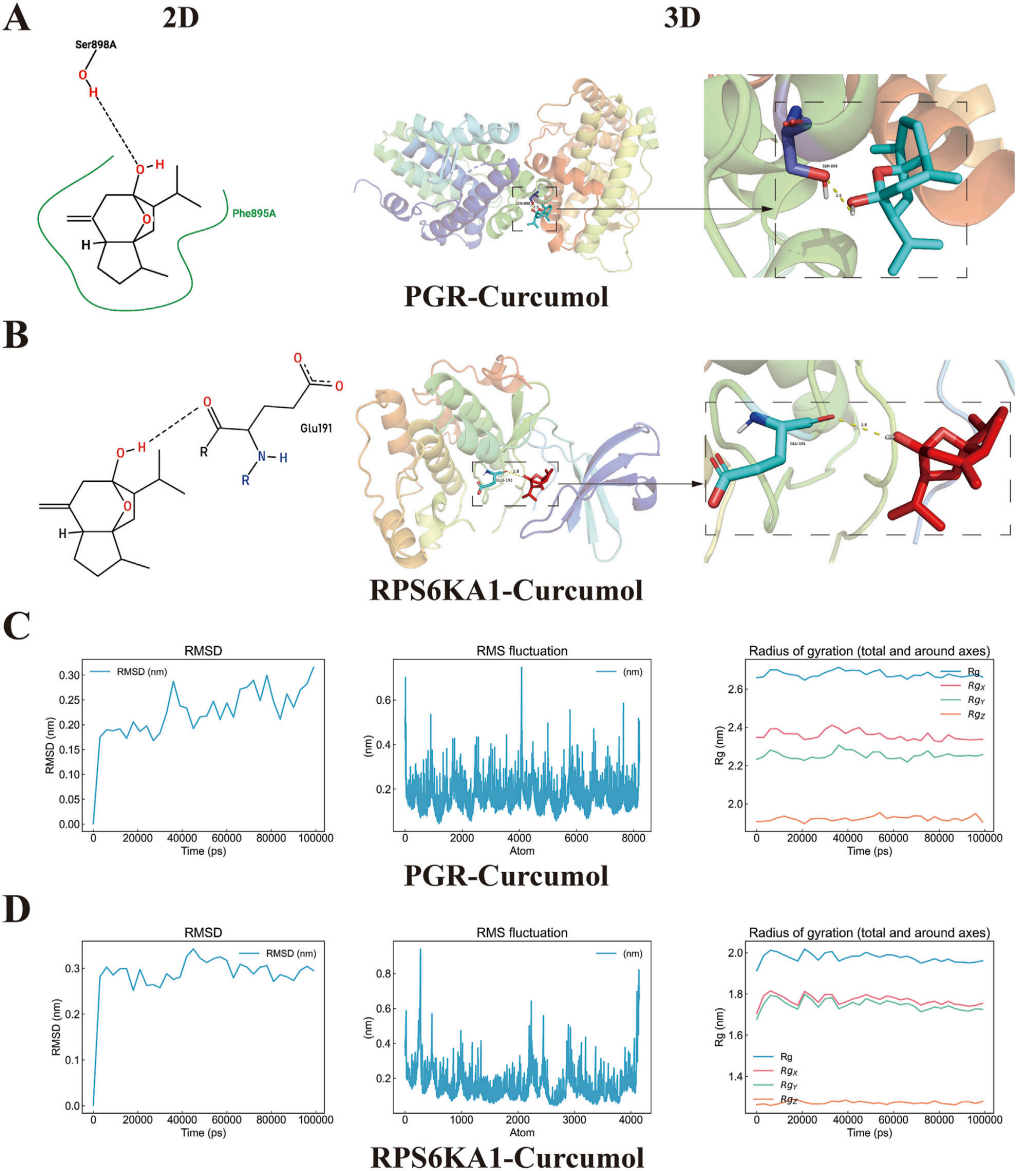
Molecular docking and dynamics simulation. **(A)** Molecular docking visualization results of curcumol-PGR. The left panel shows the 2D model, the middle panel displays the global 3D model, and the right panel presents the local 3D model, highlighting the hydrogen bonding sites between PGR and curcumol, as well as the distances between the binding sites. **(B)** Molecular docking visualization results of curcumol-RPS6KA1. **(C,D)** RMSD, RMSF, and the radius of gyration (RG) with values along the three axes (Rgx, Rgy, Rgz) for curcumol-PGR and curcumol-RPS6KA1 complexes.

Molecular docking validation was performed for the curcumol-PGR and curcumol-RPS6KA1 complexes. The interactions between curcumol and the core targets, including the formation of hydrogen bonds and the involved amino acids, were visualized in both 2D and 3D structures. Curcumol binds to the PGR protein at the SER-898 residue, forming a hydrogen bond with a bond length of 2.5 Å (Figure 7A). Additionally, curcumol interacts with the RPS6KA1 protein at the GLU-191 residue, forming a hydrogen bond with a bond length of 2.8 Å (Figure 7B). The binding energies for the complexes were as follows: curcumol-PGR, −6.6 kcal/mol; curcumol-RPS6KA1, -6.4 kcal/mol, indicating strong associations between curcumol and the core targets.

To confirm the molecular docking results of curcumol with PGR and RPS6KA1, we performed molecular dynamics simulations. The RMSD curve can reflect the volatility of molecular binding, in which PGR and RPS6KA1 proteins and curcumol have small fluctuations in the molecular simulation, but are generally stable. RMSF curve results show that when PGR and RPS6KA1 proteins bind to curcumol, their structural and functional domains have good stability, and some regions have small fluctuations, indicating that these regions have certain dynamic and flexible properties. The Rg curves of the two complexes showed consistent compactness throughout the simulation, further illustrating the effective binding of PGR and RPS6KA1 proteins to curcumol (Figures 7C,D).

### 3.9 Regulatory network of core targets

**FIGURE 8:**
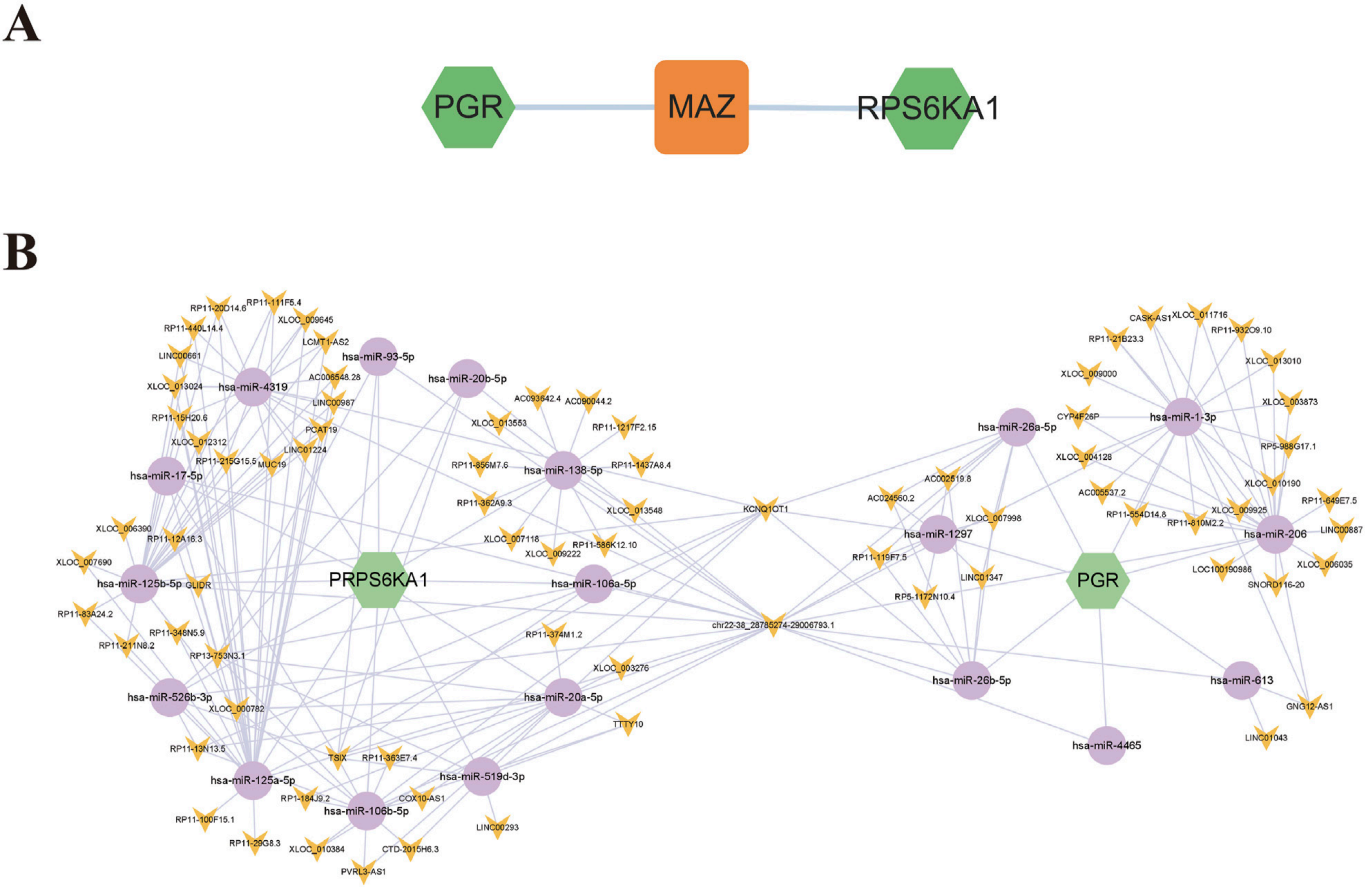
Core target regulatory network. **(A)** Transcription factor-core target network diagram: green represents the core targets, and orange represents the transcription factor. **(B)** ceRNA network diagram of core genes: green represents the core targets, purple represents the miRNAs, and yellow represents the lncRNAs.

To further investigate the potential mechanisms of PGR and RPS6KA1 in EC treatment, transcription factors (TFs), miRNAs, and lncRNAs were predicted using an online database, and a regulatory network was constructed. The transcription factor MAZ was identified as a regulator of both PGR and RPS6KA1 (Figure 8A). Additionally, a common ceRNA network involving 19 miRNAs and 79 lncRNAs was established for PGR and RPS6KA1. The hsa-miR-26 family predominantly regulates PGR, while the hsa-miR-125 family regulates RPS6KA1. Notably, KCNQ1OT1 and chr22-38_28785274–29006793.1 jointly regulate PGR and RPS6KA1 through various miRNAs, contributing to the pathogenesis of EC (Figure 8B).

### 3.10 Immunoinfiltration result

**FIGURE 9:**
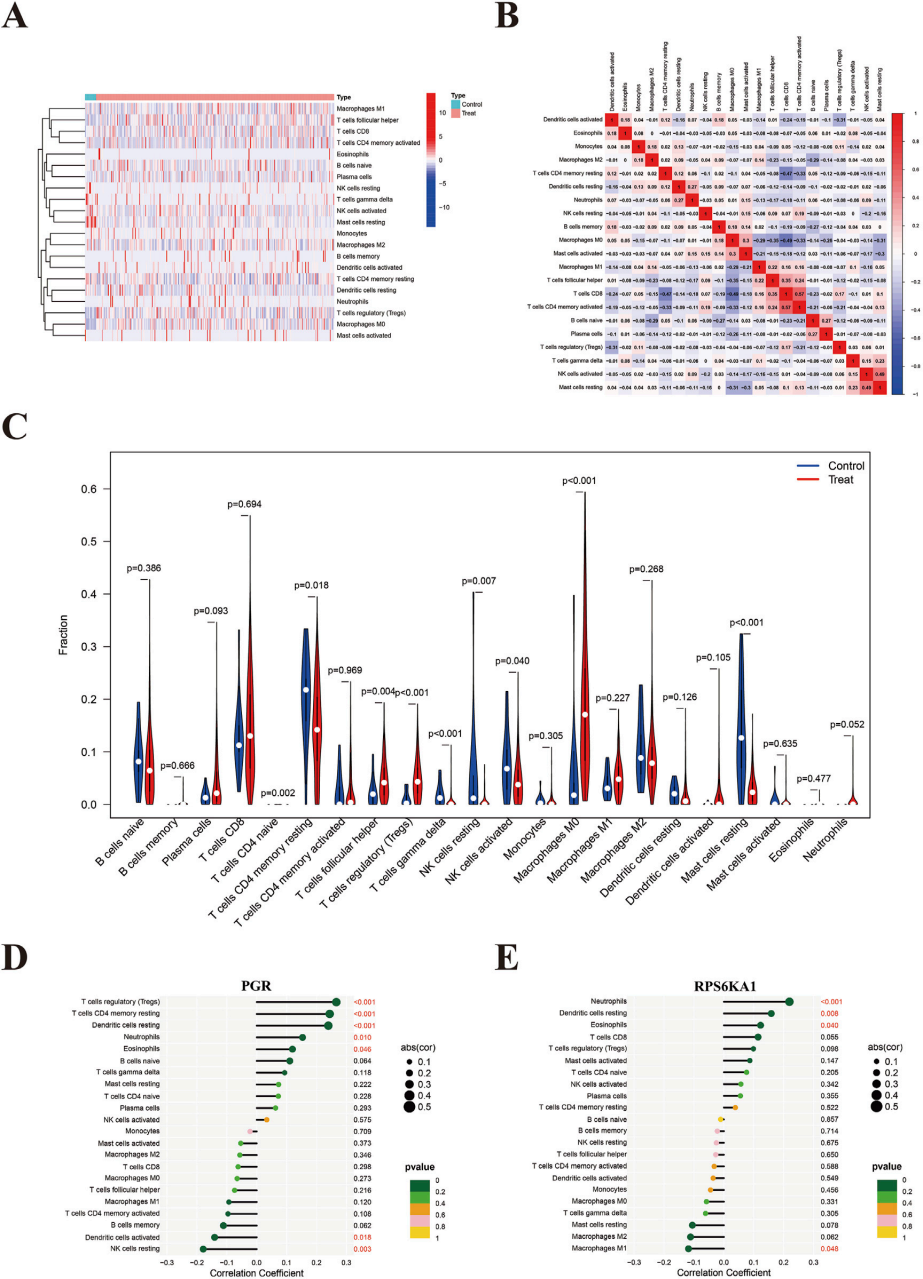
Immunoinfiltration. **(A)** Immune expression heatmap showing the differential expression of 22 immune cell types between the EC and control groups. **(B)** Immune cell correlation heatmap illustrating the relationships between 22 immune cell types; red indicates positive correlation, blue indicates negative correlation, with color intensity representing the strength of correlation. **(C)** Box plot of immune expression demonstrating the differences in the expression of 22 immune cell types between the EC and control groups. P < 0.05 indicates statistical significance, with smaller P values reflecting greater differences in expression between the groups. **(D)** Correlation between PGR and 22 immune cell types. **(E)** Correlation between RPS6KA1 and 22 immune cell types.

Heatmap and violin plot analyses revealed significant differences in the expression levels of macrophage M0, mast cells resting, T cells regulatory (Tregs), and T cells γδ between the EC and control groups, with P-values <0.001. Specifically, the expression levels of macrophage M0 and Tregs were higher in the EC group, while mast cells resting and T cells γδ were expressed at lower levels in the EC group (Figures 9A,C). The immune cell correlation heatmap indicated that most immune cells showed low correlations with each other. The highest positive correlation was observed between activated memory CD4^+^ and CD8^+^ T cells (0.57), while the highest negative correlation occurred between CD8^+^ T cells and macrophage M0 (−0.49) (Figure 9B). The lollipop plot demonstrated that PGR had the strongest positive correlation with Tregs and the highest negative correlation with resting NK cells (Figure 9D). RPS6KA1 was most positively associated with neutrophils and most negatively associated with macrophage M1 (Figure 9E).

## 4 Discussion

In recent years, the global burden of cancer has steadily increased, particularly in low- and middle-income countries. Both the incidence and mortality rates of cancer continue to rise worldwide, and this trend is expected to intensify as the global population ages (Bray et al., 2024; Global Nutrition Target Collaborators, 2025). Furthermore, the global prevalence of unhealthy lifestyles, including obesity, poor diet, physical inactivity, smoking, and alcohol consumption—especially in developing countries—has contributed to the increasing cancer burden (Blüher, 2019; Cecchini et al., 2010). Concurrently, environmental issues such as air and water pollution have become more severe, particularly in rapidly industrializing regions, further exacerbating cancer incidence and mortality (Fuller et al., 2022; Manisalidis et al., 2020).

EC, one of the most common malignancies of the female reproductive system, has shown an upward trend in both incidence and mortality worldwide in recent years, with this trend being more pronounced in developed countries (Crosbie et al., 2022; Feng et al., 2024; Mahdy et al., 2025; Makker et al., 2021). Obesity is considered a major risk factor for EC, as it leads to elevated estrogen levels, which in turn increase the risk of the disease. Developed countries such as the United States, those in Europe, and Australia report higher incidences of EC, with the incidence in the United States being approximately 25.7 per 100,000 people (Brooks et al., 2019; Crosbie et al., 2022; Feng et al., 2024; Mahdy et al., 2025; Makker et al., 2021). This is primarily associated with factors such as obesity, late marriage, delayed childbearing, and the use of hormone replacement therapy. In developing countries like China and India, while the overall incidence remains lower, it is rising due to changes in lifestyle, increasing obesity rates, and the growing aging population (Feng et al., 2024; Global Nutrition Target Collaborators, 2025; Olufadewa et al., 2021).

Natural small molecule drugs that are cost-effective, have good therapeutic outcomes, and exhibit minimal side effects have become a pressing need for patients with EC. The aim of this study is to uncover the molecular mechanisms of curcumol in the treatment of EC through multi-omics analysis.

A total of 148 curcumol-related targets were identified from the integration of five commonly used databases. Following a comprehensive screening, 2,516 DEGs related to EC were selected. After combining the datasets, 29 shared targets were identified. GO-BP analysis revealed that these 29 curcumol-EC targets were enriched in pathways such as nuclear receptor-mediated steroid hormone signaling, protein phosphorylation, and negative regulation of transcription by RNA polymerase II. This suggests that the curcumol-EC targets are involved in steroid hormone signaling and the regulation of gene expression. GO-CC and MF analyses confirmed these findings. KEGG pathway analysis further demonstrated that the 29 curcumol-EC targets were enriched in cancer-related pathways.

Through integrative analysis using univariate regression, LASSO-Cox regression, and multivariate Cox regression, PGR and RPS6KA1 were identified as core targets involved in curcumol’s anti-cancer effects in EC. The *PGR* gene encodes four isoforms of the progesterone receptor, which are involved in the regulation of gene expression and influence cellular proliferation and differentiation in target tissues (Giangrande et al., 2000). Depending on the isoform, the progesterone receptor can function either as a transcriptional activator or repressor (McDonnell et al., 1994). It also recruits the corepressor NCOR2 and activates SRC-dependent MAPK signaling upon hormone stimulation (Wen et al., 1994). The *RPS6KA1* gene encodes the ribosomal protein S6 kinase alpha-1, which positively regulates the activation of the AKT/NF-κB pathway in hepatocellular carcinoma tumorigenesis (Zhou et al., 2024). Additionally, RPS6KA1 acts as an oncoprotein in acute myeloid leukemia, promoting disease progression (Guo et al., 2024). It has also been identified as a mediator of resistance to venetoclax/azacitidine, and the inhibition of RPS6KA1 may serve as a strategy to prevent or overcome resistance in the treatment of acute myeloid leukemia (Weidenauer et al., 2023; Yu et al., 2021).

HPA validation results collectively indicated that lower expression levels of PGR and higher expression of RPS6KA1 were associated with increased EC mortality. GSEA enrichment analysis revealed that PGR and RPS6KA1 were upregulated in metabolic pathways related to matter and energy, while downregulated in developmental and cancer-related pathways.

To further explore the interactions between curcumol and its core targets, PGR and RPS6KA1, molecular docking and molecular dynamics simulations were performed. Curcumol demonstrated strong binding affinity to both PGR and RPS6KA1, confirming these as its action targets in the anti-EC effect.

To investigate the potential mechanisms of PGR and RPS6KA1 in EC treatment, regulatory RNAs were explored, and a potential regulatory network was predicted. MAZ was identified as a regulator of both PGR and RPS6KA1. Additionally, KCNQ1OT1 and chr22-38_28785274–29006793.1 were found to jointly regulate PGR and RPS6KA1 through various miRNAs, contributing to the pathogenesis of EC.

Immunoinfiltration analysis revealed that M0 macrophages and Tregs were highly expressed in EC, while resting mast cells and γδ T cells were present at lower levels. PGR showed the strongest positive correlation with Tregs and the highest negative correlation with resting NK cells. RPS6KA1 was most positively associated with neutrophils and most negatively associated with M1 macrophages. These findings suggest that PGR and RPS6KA1 play potential roles in EC treatment by modulating various immune cells.

This article explores the potential targets and mechanisms of curcumol in the treatment of EC using multi-omics approaches. However, the lack of *in vivo* and *in vitro* experimental data limits the direct validation of curcumol’s therapeutic effects on EC, thereby hindering the clinical translation of the research findings. Consequently, our next phase of research will focus on providing clear experimental evidence of curcumol’s therapeutic effects on EC through cell-based and animal studies, while also elucidating the underlying molecular mechanisms involved.

## 5 Conclusion

This study employed network pharmacology, data mining, and machine learning to integrate curcumol and EC targets. R and online databases were used to identify core targets. These core targets were validated through network pharmacological approaches, molecular docking, molecular dynamics simulations, ceRNA network analysis, clinical sample staining, and immunoinfiltration analysis. The potential molecular mechanisms by which curcumol influences EC treatment were then explored. Our findings offer valuable insights for *in vitro* studies on curcumol’s efficacy in treating EC and identify key targets for clinical applications in EC therapy. Through multi-omics analysis, we conclude that curcumol exerts its anticancer effects primarily through the core targets PGR and RPS6KA1 in the treatment of EC.

## Data Availability

The datasets presented in this study can be found in online repositories. The names of the repository/repositories and accession number(s) can be found in the article/supplementary material.
